# Posterior Fossa Progressive Multifocal Leukoencephalopathy Secondary to Rituximab

**DOI:** 10.7759/cureus.10888

**Published:** 2020-10-10

**Authors:** Mounika Guduru, Venkata Sunil Bendi, Mariana S Bebawy, Dinesh Bande, Abhishek Matta

**Affiliations:** 1 Radiology, Creighton University Medical Center, Omaha, USA; 2 Neurology, Creighton University Medical Center, Omaha, USA; 3 Family Medicine, Creighton University Medical Center, Omaha, USA; 4 Internal Medicine, University of North Dakota School of Medicine and Health Sciences, Fargo, USA

**Keywords:** pml, posterior fossa, progressive multifocal leukoencephalopathy, rheumatoid arthritis, rituximab

## Abstract

Progressive multifocal leukoencephalopathy (PML) is a rare fatal central nervous system disorder characterized by infection-induced demyelination of white matter due to the opportunistic reactivation of John Cunningham virus in an immunocompromised patient. PML is associated with many immune-mediated diseases, lymphoproliferative conditions, and immunosuppressive agents. In this case report, we present a 79-year-old female patient diagnosed with rheumatoid arthritis who developed posterior fossa PML while on rituximab. She presented with subacute cerebellar ataxia, dysarthria, and nystagmus, and her brain MRI showed right pontine and pontocerebellar lesion with diffusion restriction and heterogenous enhancement highly characteristic of PML. Though many cases of PML with rituximab were reported in the literature, our case describes a rare type of PML affecting the posterior fossa in an HIV-negative patient on rituximab.

## Introduction

Progressive multifocal leukoencephalopathy (PML) is a rare fatal central nervous system disorder characterized by infection-induced demyelination of cerebral white matter. It is due to the reactivation of John Cunningham (JC) polyomavirus in an immunocompromised patient [1,2]. JC virus primary infection often occurs during childhood or early adolescence, but the virus stays dormant in body tissues (central nervous system, kidneys, epithelium, kidneys, bone marrow, and lymph nodes) for long periods in immunocompetent individuals. Several studies in the literature reported that up to 50% of adults may be seropositive for the JC virus [3]. However, an intact immune system suppresses viral activation. In an immunocompromised state, the virus becomes reactivated, resulting in disease [4].

Immunocompromised states such as HIV infection, leukemia, lymphomas, and different malignancies are well known to be associated with PML. Several reported cases in the literature show a strong association of PML with some immunosuppressants such as natalizumab, fingolimod, dimethyl fumarate, and rituximab [4-6]. Rituximab is an anti-CD20 monoclonal antibody used in the treatment of many lymphoproliferative conditions and immune-mediated diseases such as non-Hodgkin's lymphoma, neuromyelitis optica, psoriasis, and rheumatoid arthritis [7]. The drug acts against CD20+ cells with various mechanisms of action such as antibody-dependent cytotoxicity, cell-mediated cytotoxicity, apoptosis, and direct sensitization of cells to chemotherapy [8].

## Case presentation

**Figure 1:**
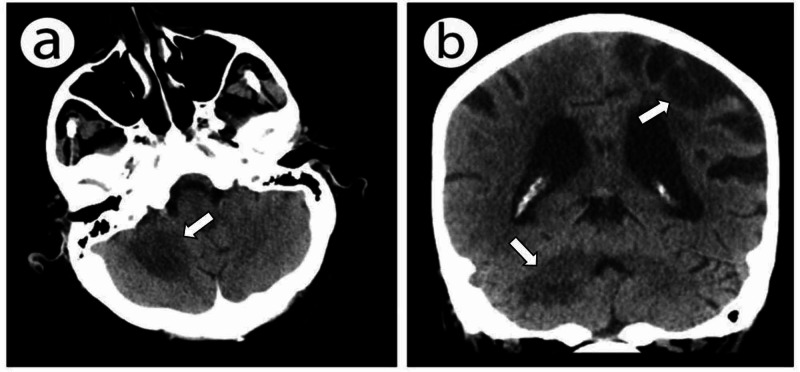
Brain CT axial (a) and coronal (b) sections showing a right cerebellar hypodense lesion with no significant surrounding edema or mass effect and left cortical and subcortical parietal encephalomalacia (on coronal section).

A 79-year-old woman with a known history of rheumatoid arthritis on long-term prednisone (5 mg daily) and rituximab (1,000 mg every six months) presented to the emergency room with multiple falls, gait difficulty, slurred speech, and confusion. She had a past medical history of hypertension and left hemispheric ischemic stroke for which she was on clopidogrel daily. On hospital admission, her vital signs were stable, and her physical examination revealed right spastic hemiparesis (a known residual from her old cerebrovascular insult), axial and appendicular ataxia, slurred dysarthria, and vertical nystagmus. Computed tomography (CT) scan of the brain showed a right pontine and right cerebellar hypodense irregular lesion without significant surrounding edema or mass effect and left parietal cortical and subcortical encephalomalacia, likely sequelae of an old vascular insult (Figure 1).

**Figure 2:**
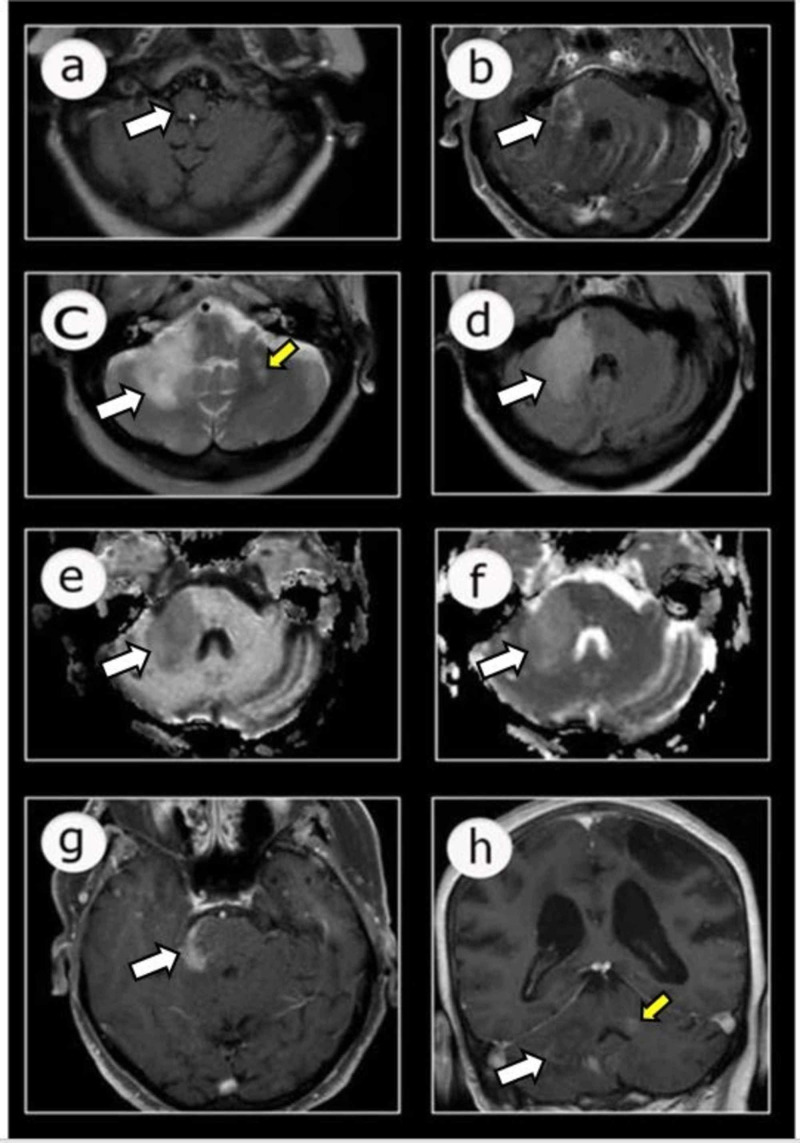
MRI of the brain with contrast showing an irregular right pontine isointense lesion on T1 (a), hyperintense signal on T2 (c), and FLAIR (d) sequences, with the extension of the lesion to the right cerebellar hemisphere showing minimal edema adjacent to the fourth ventricle. The lesion shows restriction on DWI (e) and ADC map (f) and heterogeneous enhancement at the margins (b, g, and h). Another T2 hyperintense smaller lesion is demonstrated at the left middle cerebellar peduncle and left cerebellar hemisphere (c) with heterogeneous enhancement (h). FLAIR, fluid-attenuated inversion recovery; DWI, diffusion-weighted images; ADC, apparent diffusion coefficient

Magnetic resonance imaging (MRI) with gadolinium of the brain and posterior fossa revealed the right pontine lesion (at the brachium pontis) as involving the right cerebellar white matter. The lesion was isointense on T1 and hyperintense on T2 and FLAIR (fluid-attenuated inversion recovery) sequences. The outer borders of the right pontocerebellar lesion were more defined than the inner border, and there was minimal surrounding edema, with no mass effect on the adjacent fourth ventricle. The lesions showed diffusion restriction on diffusion-weighted images (DWI) and apparent diffusion coefficient (ADC) maps. The contrast study showed heterogenous leading edge enhancement at the right pontocerebellar lesion (Figure 2). A smaller T2 and FLAIR hyperintense lesion was also noted in the left middle cerebellar peduncle, extending to the left cerebellar hemisphere with heterogeneous enhancement. These radiological features, along with the clinical features in a rituximab-treated immunocompromised patient, are highly suggestive of PML.

Her routine chemistry panel was unremarkable except for mild anemia, slightly elevated creatinine level, and hyperglycemia (Table 1).

**Table 1:** Routine laboratory tests RBC, red blood cells; WBC, white blood cells; HIV, human immunodeficiency virus; HbsAg, hepatitis B surface antigen; HCV, hepatitis C virus

Laboratory test	Admission 1	Reference range
Hemoglobin	11.5	13-15 g/dL
RBC	3.8	4.6-6.8 x 10^6^/mcL
WBC	6.4	3.6-10.3 x 10^3^/mcL
Platelets	197	140-420 x 10^3^/mcL
Blood glucose	119	70-100 mg/dL
Sodium	139	135-145 mmol/L
Potassium	4.3	3.7-5.1 mmol/L
Chloride	107	96-110 mmol/L
Bicarbonate	22	22-32 mmol/L
Ketone screen	Negative	
Blood urea	17	6-24 mg/dL
Creatinine	1.36	0.6-1.3 mg/dL
HIV	Negative	
HbsAg	Negative	
Anti-HCV	Negative	

Lumbar puncture with cerebrospinal fluid (CSF) studies and treatment options were discussed with the patient. However, the patient and her family decided to adopt a palliative approach, and the patient was discharged from the hospital to a nursing home where she died within a month.

## Discussion

In general, the incidence of PML in HIV-negative patients receiving rituximab is about 1 in every 32,000 patients [4]. Rituximab is a typical monoclonal antibody used for the treatment of many immune-mediated disorders and lymphoproliferative conditions. Despite its efficacy, it has been reported to be associated with severe adverse events particularly PML. PML is estimated to occur in around 6% of patients receiving rituximab [9], and since 1990, many case reports and case series have been published reporting the occurrence of PML with rituximab usage [7]. The patient presented in this article represents a rare case of posterior fossa PML who was on rituximab for rheumatoid arthritis. PML requires a high index of suspicion for diagnosis, and clinicians should always be on alert to include it in the differential diagnosis in immunocompromised patients, particularly those on monoclonal antibody therapy. PML is diagnosed based on clinical, radiological, and laboratory testing. Clinically, the most common three presenting symptoms are hemiparesis, visual dysfunction, and disturbed mental state [6]. However, some patients may present with posterior fossa symptoms such as ataxia, slurred dysarthria, and nystagmus [6], which are also notable in our patient. Radiologically, PML appears as white matter demyelinating single or less often as multiple lesions that predominantly affect the supratentorial structures. The lesions are characteristically large subcortical with ill-defined inner borders and well-defined outer borders that show a restriction on diffusion sequences. The lesions enhance on contrast administration typically in an open-ring pattern. However, heterogeneous patterns of enhancement were reported [10]. CSF analysis in PML might show mildly elevated proteins and white blood cells, and a high viral load. The most accurate diagnostic test of PML is a brain biopsy of the suspected lesion.

Although many cases of PML have been reported to develop in the context of rituximab therapy, the exact pathophysiological mechanism of rituximab-associated PML remains elusive. It is proposed that the rituximab-induced B-cell depletion, change in cytokine nature, and altered T-lymphocyte activity lead to reactivation of the JC virus [11]. Hematopoietic progenitor cells in the bone marrow are thought to be the site of viral latency, and when they get mobilized to the blood and central nervous system under the effect of monoclonal antibodies, they result in the hematogenous spread of the virus to the brain, activation, and development of PML [12]. As with natalizumab, the risk of PML development in patients receiving rituximab depends on the JC virus titer, the previous use of immunosuppressant medications, and the duration of use of the monoclonal antibody drug [5]. Additionally, low CD4b increases the risk of rituximab-associated PML [13]. The patient reported in this article has been on oral steroids and rituximab for years, increasing her risk of PML. As PML can be fatal, patients receiving rituximab should be meticulously monitored. Regular follow-up of CD4b is essential, and abrupt discontinuation of rituximab is fundamental to reduce mortality if PML is suspected [4]. The FDA has granted Orphan Drug designation to NT-I7 (efineptakin alfa) for the treatment of PML. Plasma exchange to remove the circulating drug as well as stopping the offending drug represent the treatment of choice for these cases [6].

## Conclusions

As PML can be fatal, patients receiving rituximab should be meticulously monitored. Regular follow-up of CD4b is essential, and abrupt discontinuation of rituximab is fundamental to reduce mortality if PML is suspected. Although there is no definitive evidence that FDA approved the medication for PML to date, plasma exchange to remove the circulating drug as well as stopping the offending drug represent the treatment of choice for these cases.
